# Hospital at home: The role for clinical pharmacy in an innovative acute care model in British Columbia

**DOI:** 10.1177/17151635211045951

**Published:** 2021-09-24

**Authors:** Morgan E. Patrick, Curtis K. Harder, Sean P. Spina

**Affiliations:** Faculty of Pharmacy and Pharmaceutical Sciences, University of Alberta, Edmonton, Alberta; Victoria General Hospital, Vancouver Island Health Authority; Faculty of Pharmaceutical Sciences, University of British Columbia, Vancouver, BC; Royal Jubilee Hospital, Vancouver Island Health Authority; Faculty of Pharmaceutical Sciences, University of British Columbia, Vancouver, BC

## Introduction

In November 2020, Island Health, with the support of the BC Ministry of Health, introduced Hospital at Home (HaH), an innovative model of acute care that provides hospital-level treatment and services to patients in their own homes. The combination of in-person and virtual supports allows patients to receive safe and effective care from acute care health care providers. Despite being at home, patients are “admitted” to the hospital and remain under the care of a hospital-based team.

The pharmacy department was identified as a major stakeholder at idea inception, given the target patient population and the requirement for medication therapy. Distribution of medications to patients in their homes brought with it obvious challenges. However, given the acuity of the patients anticipated to receive care through this model, questions quickly arose about how the delivery of clinical pharmacy services that hospital inpatients rely on could be included in the HaH model. Planning for inclusion of a clinical pharmacist on the HaH team required a review of clinical pharmacy activities that could be undertaken in a hybrid in-person/virtual model.

The objective of this article is to elaborate on the role of the HaH clinical pharmacist and outline how the role’s impact is being evaluated. A brief overview of the program and the current supporting evidence will be provided.

## Supporting evidence for HaH programs

HaH has been successfully implemented in Australia, England, Scotland, Spain and France. Many studies, including 4 Cochrane reviews, indicate that HaH provides patients similar or better care than traditional brick-and-mortar hospitals.^1-4^ This patient-centred approach has resulted in fewer hospital readmissions,^4^ shorter lengths of hospital stay,^1^ increased patient and caregiver satisfaction,^1^ lower risk of institutional living within 1 year^1^ and fewer behavioural issues among patients with dementia.^2^ In 2020, a narrative literature review of 29 English-language articles focusing on the health and economic benefits of HaH programs compared with hospital facility admissions for patients with chronic obstructive pulmonary disease and heart failure showed that HaH is a safe and cost-effective model that will increase hospital bed capacity and allow patients to receive care in the setting they prefer.^5^

Stakeholder-reported experience and outcome measures have also been studied. In 2019, a qualitative study embedded within a randomized controlled trial assessed the positive drivers and potential barriers to implementation of a HaH program.^6^ The study population captured a small (*n* = 89) but broad range of stakeholders, including patients, caregivers, doctors, specialist nurses and managers. Semi-structured interview questions were developed within a HaH pilot project and refined using patient feedback. The transcripts were analyzed and coded by 3 independent reviewers using a combined inductive-deductive method known as “thematic-construct analysis.” The authors outline positive drivers to include availability of home comforts; maintained independence; improved sleep and nutrition; perception of faster recovery; ease of visits by friends and family; confidence in the continuity of care; feelings of safety, reassurance and appreciation; and increased privacy for clinical assessments and personal care. Potential barriers include fear of being alone and privacy issues associated with not wanting visitors to enter the home.

## Island Health’s HaH prototype

A HaH prototype was launched in November 2020, based at the Victoria General Hospital in Victoria, British Columbia. The program was expanded to include a second site at the Royal Jubilee Hospital, 13 km away. The program functions as an additional medicine ward for both hospitals, with patients being registered as inpatients. The program offers around-the-clock substitutive hospital-level care to eligible and consenting patients within a specified catchment radius around the hospital. The team is composed of hospitalist physicians, a clinical pharmacist, acute care nurses, clinical nurse leaders, unit clerks and a hospital liaison nurse. The clinical pharmacist component began with service 7 days per week during daytime hours (1.4 full-time equivalents).

## Supporting evidence for inclusion of clinical pharmacy within HaH teams

Inclusion of a hospital pharmacist within Island Health’s prototype required significant deliberation because of limited supporting evidence. A literature review of the Cochrane Library, PUBMED, EMBASE and Google Scholar identified 1 conference abstract published in 2020 of a retrospective study titled “Pharmacist Interventions in a Hospital at Home Unit.”^7^ The review included all patients admitted to a HaH unit in Portugal between December 2018 and August 2019. The pharmacist interventions were classified by the type and reason for intervention and therapeutic recommendation. In total, 80 pharmacist interventions occurred for 53 patients out of 425 patients admitted to HaH. Pharmacists often intervened with the numerous medications prescribed to patients, 63% of whom were receiving more than 10 medications. The most common pharmacist activities included pharmacokinetic monitoring (45%), medication review (28%) and prescription validation (23%). The recommendations often pertained to making dose adjustments for subtherapeutic medications and changing prescribed medications. The authors reported an acceptance rate of 96.3% for the pharmacist recommendations and concluded that participation of a pharmacist in a HaH team positively affects patient safety. With only 1 published study assessing the role that clinical pharmacy has in a HaH program, more research is warranted. For this reason, the Island Health Pharmacy–led HaH research team is committed to evaluating the role of the clinical pharmacist and including outcome measures pertaining to efficacy, safety and stakeholder experience.

## Role of clinical pharmacy within Island Health’s HaH prototype

Planning for the inclusion of a clinical pharmacist involved reviewing conventional hospital-oriented clinical pharmacy activities to determine how to incorporate them into the HaH model. For guidance, we turned to the Canadian Society of Hospital Pharmacists (CSHP) to examine our national society’s stance on the role that clinical pharmacy might have in this novel setting. In 2016, CSHP published 56 position statements that articulate the role pharmacy has in the provision of patient care within health care organizations.^8^ These statements were reviewed for applicability to HaH and the result was a defined care process that outlines pharmacy tasks at admission, during admission and at discharge.

Although many of the identified tasks are consistent with those conducted by clinical pharmacists in the hospital setting (Table 1), a few distinct differences were identified. For example, HaH patients assume a greater responsibility for their own care. As such, inclusion of the patient in establishing goals of therapy, provision of appropriate education on medication administration and safety and efficacy monitoring are elevated in priority. It is also critical that clinical pharmacists be able to adapt their communication style and method to suit patients’ needs. Particularly at implementation, while care delivery processes are refined to suit the HaH setting, we expect the clinical pharmacist will serve an essential role in addressing logistical issues related to the delivery of medication therapies, previously provided only in the hospital setting, to the HaH setting.

**Table 1:** Responsibilities of the HaH pharmacist

● Support medication reconciliation on admission and discharge through consultation with admitting physician. ● Enter and verify new and changed medication orders for admitted patients in the electronic health record. ● Liaise with inpatient pharmacy department to ensure daily dispensing of required medications. ● Engage patients in discussion about medication-specific outcomes, as appropriate. ● Clinically assess patients for the following: ○ Drug therapy issues that could have contributed to and/or complicated admission ○ Appropriateness, effectiveness and safety of medications ordered during admission ○ Ability to adhere to medication prescribed during HaH admission (e.g., intravenous antibiotics requiring more than once-daily frequency of administration) ● Identify, resolve and document drug therapy issues. ● Document and implement pharmacy care plans, including schedule for monitoring and follow-up. ● Provide medication education to patients, families and caregivers, as necessary. ● Liaise with community pharmacy to ensure safe and efficient transfer of pharmacy care. ● Assist with necessary special approvals to ensure continuity of pharmacy care (e.g., provincial special authority program).

## Evaluation of the clinical pharmacy role within Island Health’s HaH prototype

The research team is assessing the clinical pharmacist’s contributions to patient care using clinical pharmacy key performance indicators (cpKPIs), which are recognized by Canadian hospital pharmacists.^9^ These cpKPIs represent activities carried out by clinical pharmacists and are known to affect patient outcomes, such as hospital readmissions. The cpKPIs include performing medication reconciliation on admission, participating in interprofessional patient care rounds, delivering and initiating pharmaceutical care plans, identifying and resolving drug therapy problems, providing patient medication education during hospital stay and at discharge, performing medication reconciliation at discharge and delivering all activities through collaborative patient care. Data are also being collected to quantify the clinical pharmacist’s involvement in patient care. This may include how many times the pharmacist reviews patient medication lists, completes special authorization requests, makes phone or video calls to the patient or visits the patient in the home. The research team is also assessing whether the allotment of 1.4 full-time equivalents for pharmacist staffing is sufficient to support the needs of the patients. Understanding that health care provider and patient experience is imperative when launching a new program, the research team is using a variety of methods to gain insight into these experiences. Questionnaires are conducted verbally over the phone with patients and caregivers after discharge to assess their respective experiences. Additionally, focus groups and interviews are conducted with clinical staff and leaders on a regular basis to formally obtain their feedback. As data are collected, refinements are being made to the program and future publications will provide more information about what the research team learns about the role that clinical pharmacy has within a HaH program.

The pharmacy profession continues to evolve in accordance with a growing need for specialized medication knowledge. Although preparing and dispensing medications are fundamental to pharmacy care delivery, the provision of patient-centred, outcome-focused care is essential.^10^ Pharmacists offer an in-depth understanding of how medications work and have the experience to address complications that may result from complex medication regimens. The introduction of a novel care model brings with it the opportunity to carefully consider what the determinants for success will be from the outset. Without question, the HaH model will stretch our conventional thinking about delivering great care, including all that is required for the provision of safe and effective medication therapy. Clinical pharmacists have demonstrated that their contributions are invaluable to patient care in the hospital setting, unique from but complementary to those of other health disciplines. As such, it naturally follows that the clinical pharmacist on the HaH care team will *begin* his or her role as an essential member. Harkening back to the adage “the absence of evidence is not the evidence of absence (of evidence),” we expect that the evidence to support our conviction will follow. ■
